# In question: the scientific value of preclinical safety pharmacology and toxicology studies with cell-based therapies

**DOI:** 10.1038/mtm.2014.26

**Published:** 2014-07-16

**Authors:** Christiane Broichhausen, Paloma Riquelme, Norbert Ahrens, Anja K Wege, Gudrun E Koehl, Hans J Schlitt, Bernhard Banas, Fred Fändrich, Edward K Geissler, James A Hutchinson

**Affiliations:** 1 Division of Experimental Surgery, Department of Surgery, University Hospital Regensburg, Regensburg, Germany; 2 Department of Transfusion Medicine, Institute for Clinical Chemistry, University Hospital Regensburg, Regensburg, Germany; 3 Department of Obstetrics and Gynaecology, University Hospital Regensburg, Regensburg, Germany; 4 Nephrology Division, Department of Internal Medicine II, University Hospital Regensburg, Regensburg, Germany; 5 Institute for Applied Cell Therapy, University Hospital of Schleswig-Holstein, Campus Kiel, Kiel, Germany

## Abstract

A new cell-based medicinal product containing human regulatory macrophages, known as Mreg_UKR, has been developed and conforms to expectations of a therapeutic drug. Here, Mreg_UKR was subjected to pharmacokinetic, safety pharmacology, and toxicological testing, which identified no adverse reactions. These results would normally be interpreted as evidence of the probable clinical safety of Mreg_UKR; however, we contend that, owing to their uncertain biological relevance, our data do not fully support this conclusion. This leads us to question whether there is adequate scientific justification for preclinical safety testing of similar novel cell-based medicinal products using animal models. In earlier work, two patients were treated with regulatory macrophages prior to kidney transplantation. In our opinion, the absence of acute or chronic adverse effects in these cases is the most convincing available evidence of the likely safety of Mreg_UKR in future recipients. On this basis, we consider that safety information from previous clinical investigations of related cell products should carry greater weight than preclinical data when evaluating the safety profile of novel cell-based medicinal products. By extension, we argue that omitting extensive preclinical safety studies before conducting small-scale exploratory clinical investigations of novel cell-based medicinal products data may be justifiable in some instances.

## Introduction

Transferring immunoregulatory cells from a tolerant donor to nontolerant individual as a means of establishing tolerance in the recipient is a common technique in experimental immunology, but its clinical application is only now receiving serious attention.^1^ Several classes of immunoregulatory cells are currently being developed as adjunct immunosuppressive agents for use in solid organ transplantation, including several types of regulatory T cells^2–4^ and suppressive myeloid cells.^5–8^ One particularly promising candidate cell type is the human regulatory macrophage.^9^ The regulatory macrophage (M reg) phenotype reflects a unique state of macrophage differentiation, distinguished from macrophages in other activation states by its mode of derivation, robust phenotype, and potent suppressor function.^10^ M regs prevent mitogen-stimulated T cell proliferation *in vitro* through IFN-γ–induced indoleamine 2,3-dioxygenase activity, as well as mediating a contact-dependent deletion of activated T cells.^11^ In addition, M regs drive the development of activated induced regulatory T cells (iTreg) that, in turn, suppress the proliferation of effector T cells and inhibit the maturation of dendritic cells (Walter *et al*. unpublished data). Therefore, it is speculated that when M regs are administered to a transplant recipient, they initiate a feed-forward loop of allospecific regulation.

M reg-containing cell preparations have been administered to a total of 19 kidney transplant recipients in a series of case studies and two early-phase clinical trials.^12–15^ While these pilot studies do not provide conclusive evidence of the safety or efficacy of M reg treatment in renal transplantation, they do demonstrate the feasibility of delivering donor-derived M reg therapy to renal transplant recipients.^16^ A further two living-donor kidney transplant recipients have now been treated with ~8.0 × 10^6^ cells/kg bodyweight (BW) of highly purified donor-derived M regs.^11^ These patients are now more than 5 years posttransplantation with stable renal function, receiving only low-dose tacrolimus monotherapy as maintenance immunosuppression. A newly developed therapeutic cell product containing M regs (known as Mreg_UKR) conforms to our expectations of a clinically applicable drug product. A further clinical trial of M reg therapy in living-donor renal transplantation is now authorized within the framework of the ONE Study (http://www.onestudy.org). This trial (ONEMreg12; EudraCT Nr.: 2013-000999-15; ClinicalTrials.gov: NCT02085629) aims to treat 16 patients with donor-derived Mreg_UKR at a dose of 2.5–7.5 × 10^6^/kg BW under cover of 500 mg/day MMF on day 7 prior to surgery.^17^

Although cell-based medicines are quite different in nature from chemically synthesized drugs, many of the same general considerations apply to their clinical use. In order to use any therapeutic agent safely and effectively, clinicians must know about its pharmacological properties and how these predict efficacy and safety in individual patients.^18^ Specifically, clinicians must know about the pharmacokinetics (*i.e.*, absorption, tissue distribution, metabolism, and elimination) and therapeutic dose-range of a drug, as well as having an understanding of its mechanism of action and potential adverse effects. These clinical considerations are now reflected in European Law^19–21^ and guidance issued by the European Medicines Agency.^22^

Current European Medicines Agency guidelines on cell-based medicinal products (CBMPs) stipulate that novel cell products must be subjected to conventional toxicological and safety pharmacology studies.^23^ Toxicology studies are principally concerned with defining the relationship between drug exposure and its adverse effects, usually taking structural changes to tissues upon postmortem examination as their major endpoint. Accordingly, a key objective of toxicological studies is defining the maximum tolerated dose of a drug in single and repeat doses. In contrast, safety pharmacology studies seek to predict whether a drug is likely to be found unsafe when administered to patients at therapeutic doses and thereby aim to prevent such occurrences. Within this remit, safety pharmacology studies try to predict the possible occurrence of rare adverse events.^24^ In practical terms, this entails showing whether a drug is safe or unsafe using a core battery of pharmacological tests to assess adverse reactions affecting the central nervous, cardiovascular, respiratory, and other organ systems.^25^ A general requirement for toxicological and safety pharmacology studies in the drug development process are now codified in International Conference on Harmonisation (ICH) guidelines (CPMP/ICH/539/00).^26^

This article presents the results of preclinical studies into the pharmacokinetics, acute and chronic toxicity, carcinogenicity, and safety pharmacology of Mreg_UKR, which were presented to the German National Competent Authority, the Paul Ehrlich Institute (http://www.pei.de) as part of a successful application for authorization to conduct the *ONEmreg12* clinical trial. As an academic research group with no prior experience in drug development, we consulted an independent regulatory affairs advisor to devise a preclinical safety testing strategy that complied with all relevant regulatory obligations. This strategy was endorsed by the Paul Ehrlich Institute at a *scientific advice* meeting. On the basis of this advice and our own interpretation of European Medicines Agency guidelines,^23^ a clinical trial application was lodged with the competent authority, which incorporated the pharmacokinetic, acute toxicity, chronic toxicity, and carcinogenicity studies presented in this article. This application was initially rejected owing to shortcomings in the clinical protocol and all three principal sections of the Investigational Medicinal Product Dossier. Notably, the Authority commented on the inadequacy of our biochemical and clinical investigations of mice treated with Mreg_UKR; hence, the safety pharmacology studies presented in this article were performed. In response to its critique, a revised clinical trial application was submitted to the Paul Ehrlich Institute and was granted approval.

The preclinical studies described in this article found no evidence of acute or chronic adverse reactions to therapeutic doses of Mreg_UKR; accordingly, they present no impediment to the further development of Mreg_UKR as a pharmaceutical agent. However, this work brings into question the relevance of applying animal-into-animal (homologous) and human-into-animal (heterologous) safety testing strategies to CBMPs. In particular, this article illustrates how easily preclinical pharmacokinetic and safety pharmacology studies could lead to false conclusions about the probable pharmacological properties of CBMPs in human recipients. Hence, a major conclusion of this work is that previous clinical experience from exploratory trials should be afforded far greater importance in assessing the potential clinical risk profile of Mreg_UKR therapy than preclinical animal experiments. By extension, we argue that there is a case for conducting small-scale exploratory clinical studies of novel CBMPs without extensive preclinical safety investigation, especially when similar CBMPs were already administered patients without adverse effects.

## Results

### Tissue distribution and survival of Mreg_UKR in NSG mice

The eventual distribution of Mreg_UKR after intravenous infusion reflects their passive and active migration to different sites, their engraftment in those tissues, as well as their death and elimination. To track the survival and tissue distribution of M regs *in vivo*, human cells were injected into NSG mice and their presence in blood, spleen, bone marrow, liver, and lungs was assessed by flow cytometry on days 1–7 postinjection. Recipients were randomized to two treatment groups, which received either 5 × 10^6^ viable M regs or vehicle-only. Prior to detection by flow cytometry, M regs were enriched from dissociated tissues by positive selection of CD11b^+^ cells with magnetic selection beads. Notably, this method of detection gives only qualitative information about the presence or absence of M regs in a tissue. M regs present in mouse tissues were identified by flow cytometry as living human CD45^+^ cells that coexpressed CD11b and HLA-DR. In previous work, we have shown that human M regs are homogeneously CD45^+^ CD11b^+^ HLA-DR^+^ CD14^−/low^ and CD16^−/low^ in phenotype.^11^ To assess the stability of the M reg_UKR phenotype after administration to mice, expression of CD14 and CD16 by living M regs recovered from mouse tissues was also investigated.

**Figure 1:**
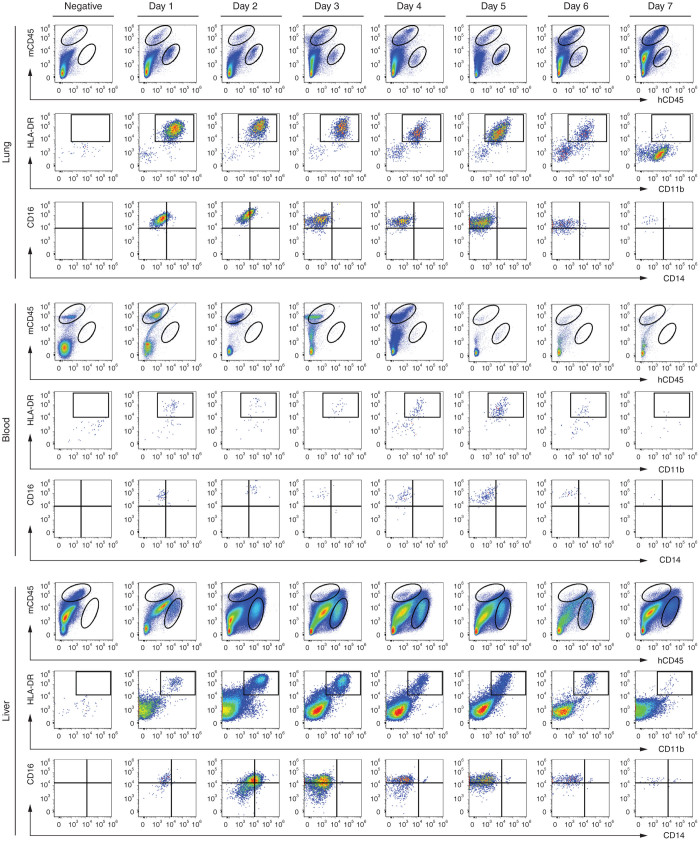
Distribution and fate of Mreg_UKR in NSG mice. Recipient mice were given 5 × 10^6^ viable M regs or vehicle-only by slow intravenous injection. The tissue distribution of M regs was then assessed on days 1 to 7 post-injection by flow cytometry. Human M regs defined by expression of human CD45, CD11b and HLA-DR were detected in lung, blood and liver at all timepoints. Although the engrafted M regs remained CD14^-^, CD16 expression was regained by day 1. Data are representative of at least two animals per timepoint from at least two independent experiments.

Human M regs were detectable in lung, blood, and liver for up to 7 days postinfusion (Figure 1). It was not possible to reliably detect human M regs at any time point in spleen and bone marrow, either because human M regs were not present or because they were indiscriminable from the large populations of mouse macrophages present in those tissues. M regs retained their CD11b^+^ HLA-DR^+^ CD14^−/low^ phenotype throughout the 7-day observation period. In contrast, M regs upregulated CD16 expression within 1 day of infusion, which possibly reflects the absence of human immunoglobulins in NSG mice.^27^

### Clinical observation of NMRI-nude mice after Mreg_UKR injection

Fifteen age-matched, male NMRI-nude mice were randomized to three treatment groups of five animals. NMRI-nude mice are congenitally athymic, so are effectively T-cell deficient, but produce functionally normal B cells. Hence, NMRI-nude mice were chosen for safety pharmacology studies because they are incapable of T cell-mediated rejection of xenogeneic cells, while still being able to mount innate immune and IgM responses that might contribute to adverse reactions.

Recipient mice were anesthetized and fully anticoagulated with 60 IU heparin prior to slow (30–180 seconds) intravenous injection of M regs via the tail vein. For injection, M regs were suspended in Ringer’s lactate solution plus 5% human albumin. Mice in treatment group 1 received 1 ml vehicle-only. Mice in groups 2 and 3 received 10^6^ or 10^7^ viable M regs suspended in a volume of 1 ml, respectively. These cell doses corresponded to 34.0 ± 3.1 × 10^6^ cells/kg BW and 356.8 ± 31.9 × 10^6^ cells/kg BW, respectively. Recipient mice were closely observed for 3 hours following Mreg_UKR injection to assess their clinical responses, particularly with regard to respiratory rate and rhythm. Over the subsequent 7 days, recipient mice were checked daily for constitutional signs of adverse drug reactions and BW changes were recorded.

**Figure 2:**
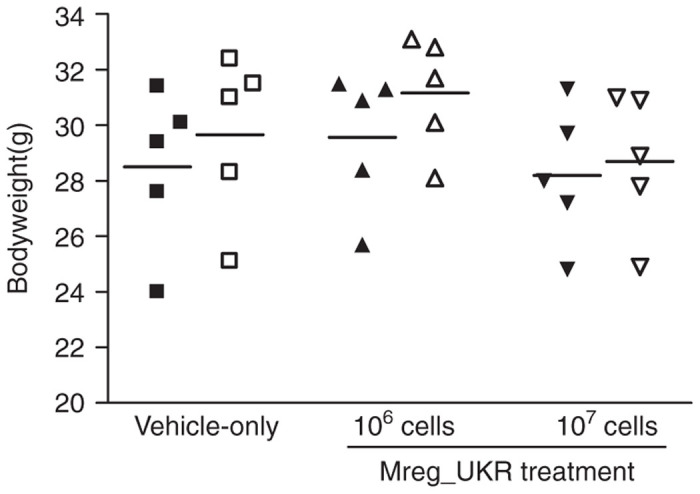
Weight gain in NMRI-nude mice was unaffected by human regulatory macrophages (Mreg_UKR) treatment. NMRI-nude mice were allocated to three groups of five animals. Mice received injections of either 10^6^ or 10^7^ viable human M regs resuspended in Ringer’s lactate solution plus 5% human serum albumin and 60 U heparin, or were given a vehicle-only injection. No significant difference in weight gain was observed over a 7-day observation period after Mreg_UKR administration. (Filled symbols indicate bodyweight on day 0; unfilled symbols indicate bodyweights on day 7.)

No acute adverse reaction to Mreg_UKR doses of 10^6^ or 10^7^ cells was detected. In particular, no deaths occurred following intravenous cell infusion, no change in respiratory rate or rhythm was detected, and no dyspnea, hemoptysis, or cyanosis was observed. This is perhaps a reassuring result because one theoretical concern with infusion of M regs, which have a diameter of 15–30 μm, is obstruction of pulmonary vessels by single cells or cell aggregates.^18^ Recipient mice in all groups recovered from general anesthesia within 30 minutes, and none showed signs of distress upon waking. At 3 hours postinfusion, mice from all treatment groups were normally active, and an abbreviated clinical examination revealed no respiratory or neurological abnormalities. No significant difference in weight gain between treatment groups was observed over the 7-day study (Figure 2). No delayed reactions, as assessed by changes in behavior or constitutional signs, were observed over the 7-day follow-up period, and no deaths occurred.

At 7 days postinfusion, mice in all treatment groups were normally active and showed no grossly unusual behavior. Specifically, recipient mice were examined using an adaptated version of Irwin’s comprehensive observational assessment, which assesses behavioral, neurological, and autonomic responses to drug treatments.^28^ No clinically relevant differences in performance between treatment and control groups were detected (Table 1). No signs of dermatological disease were observed, although a few animals in each treatment group bore bite-marks. There was no sign of disturbed bowel function or rectal prolapse in any of the animals. Respiratory rate was not different between the treatment groups and respiratory rhythm was regular in all recipients (Table 1).

### Postmortem examination of NMRI-nude mice on day 7 after Mreg_UKR injection

Upon thoracotomy under anesthesia, heart rate was not significantly different between the treatment groups and cardiac contraction was organized and regular in all recipients (data not shown). The lungs appeared pink and uniformly well perfused. No gross pathological changes were evident in the Mreg_UKR-treated or control mice. Specifically, there was no sign of myocardial infarction or distension of the atria, ventricles or pulmonary arterial trunk in any animals. The abdomen contained no ascites, blood, tumors, or adhesions in any recipients or controls. The large and small intestines, spleen, urinary bladder, kidney, liver, pancreas, kidneys, and great vessels of all animals appeared grossly normal. No other gross abnormalities were noted. Organ weights were not significantly different between Mreg_UKR-treated and untreated recipients (Table 2).

**Figure 3:**
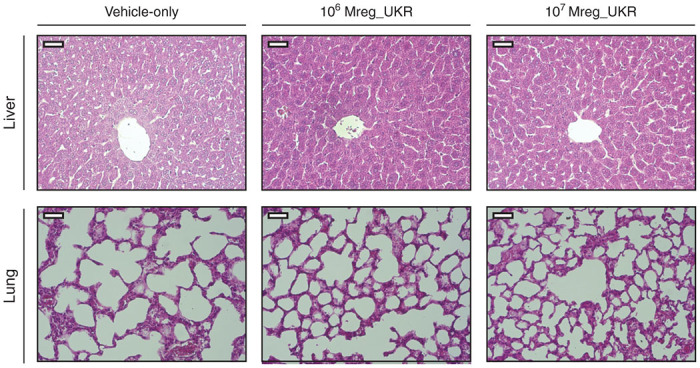
Histopathological survey of tissues from human regulatory macrophages (Mreg_UKR)-treated NMRI-nude mice. NMRI-nude mice were allocated to three groups of five animals. Mice received injections of either 10^6^ or 10^7^ viable human M regs resuspended in Ringer’s lactate solution plus 5% human serum albumin and 60U heparin, or were given a vehicle-only injection. Seven days after Mreg_UKR administration, recipient mice were killed and tissues were harvested for histopathological examination. Three micrometer sections were cut from paraformaldehyde-fixed, paraffin-embedded tissues and then stained with hematoxylin and eosin. No tissue pathology associated with Mreg_UKR administration was observed. Representative images of liver and lung, the tissues in which M regs principally accumulate, are shown (Bar = 50 μm).

Histological sections of brain, lung, heart, liver, spleen, duodenum, right colon, and kidney were prepared from paraformaldehyde-fixed, paraffin-embedded tissues and stained with hematoxylin and eosin. Tissue sections were evaluated blindly. No microscopic tissue pathology associated with Mreg_UKR administration was observed (Figure 3).

### Biochemical investigation of NMRI-nude mice on day 7 after Mreg_UKR injection

**Figure 4:**
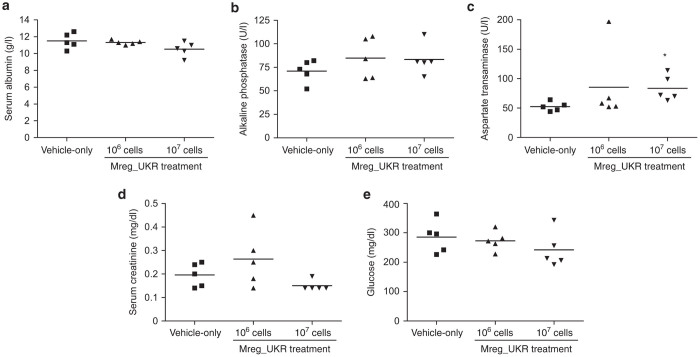
Biochemical investigation of NMRI-nude mice after human regulatory macrophage (Mreg_UKR) treatment. NMRI-nude mice were allocated to three groups of five animals. Mice received injections of either 10^6^ or 10^7^ viable human M regs resuspended in Ringer’s lactate solution plus 5% human serum albumin and 60 U heparin, or were given a vehicle-only injection. Seven days after Mreg_UKR administration, recipient mice were killed and serum levels of albumin, alkaline phosphatase, aspartate transaminase, creatinine and glucose were investigated. No signficant differences were observed between treatment groups, except for a marginal increase in aspartate transaminase levels in mice treated with 10^7^ M regs (Kruksall–Wallis test; 10^7^ Mreg_UKR versus vehicle-only, *P = 0.036).

Given that Mreg_UKR distributed primarily to liver, recipient mice were investigated for markers of liver injury: serum albumin (Figure 4a) and alkaline phosphatase (Figure 4b) levels were not significantly different between treatment groups; however, a marginal increase in serum aspartate transaminase (AST) was observed in group 3 (Figure 4c). The biological relevance of such a small difference serum AST levels is presently unknown. Notably, among the 21 patients treated with M reg-containing cell products, who are all more than 5 years posttreatment, no incidents of disturbed liver function tests were reported. Serum alkaline phosphatase is also a marker of increased bone resorption and serum albumin levels are typically reduced as part of the acute phase response; therefore, no biochemical evidence was found of increased bone turnover or systemic inflammation caused by Mreg_UKR. To investigate the possibility that Mreg_UKR affect renal function by embolising (in the form of individual cells, cell aggregates, dead cells, or immune complexes) to renal glomeruli, serum creatinine levels were measured as an indicator of filtrative capacity: no differences were observed between treatment groups (Figure 4d). Glucose levels are a sensitive, albeit very unspecific, parameter to screen for adverse drug reactions: Hypoglycemia might result from sepsis, disturbances of the hypothalamic–pituitary–adrenal axis resulting in reduced glucocorticoid production or disturbed insulin production (or IGF-2 production); hyperglycemia may result from pituitary, adrenal, or pancreatic dysfunction or could indicate ischemic disease or infections. No significant changes in glucose levels were observed (Figure 4e).

### Immunogenicity of allogeneic mouse M regs in immunocompetent recipients

**Figure 5:**
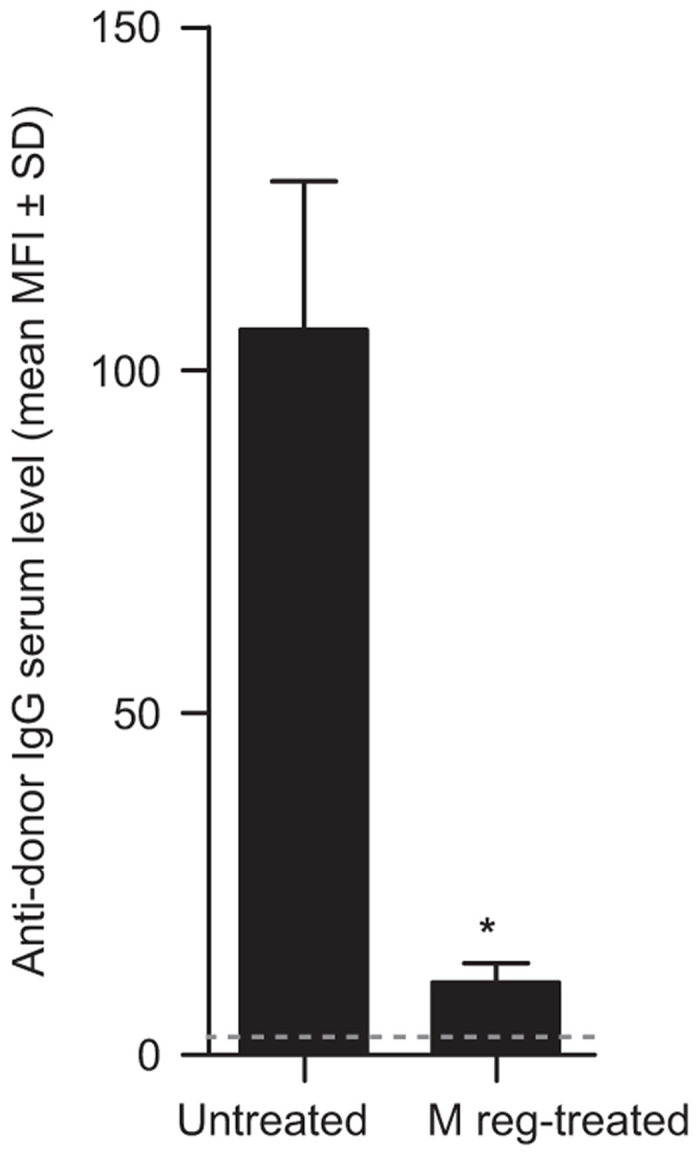
Assessing the immunogenicity of allogeneic mouse M regs in mice. BALB/c mice were either treated with 5 × 10^6^ C3H-derived M regs (*n* = 4) or received no cells (*n* = 3). Eight days later, all mice were given a heterotopic heart transplant from a C3H donor. None of the transplants failed before day 7. On day 7, sera were harvested from all mice for measurement of antidonor IgG and IgM antibody titers by flow cytometry crossmatch. Mice treated with M regs 8 days before transplantation registered significantly lower levels of antidonor IgG than untreated controls (Mann–Whitney *U*-test; *P < 0.001). Gray line indicates limit of detection..

To formally assess the risk of humoral sensitization by M regs, donor-specific anti-major histocompatibility complex class I antibody responses were measured in BALB/c mice that received C3H cardiac allografts after preoperative treatment with donor strain-derived M regs. As previously published, no accelerated allograft lost was observed in the M reg-treated recipients, indicating that M reg administration on day 8 prior to transplantation did not sensitize recipients.^10^ Here, sera were harvested from mice 7 days after heart transplantation, and their alloantibody content was measured by flow cytometry cross-match. Consistent IgG responses were detected in transplanted mice without M reg treatment; in contrast, mice treated with 5 × 10^6^ donor-derived M regs 8 days prior to transplantation had significantly lower levels of antidonor IgG (Figure 5). No antidonor IgM response was detected in either the control or M reg-treated group (data not shown). Therefore, there is no evidence that intravenous injection of allogeneic mouse M regs caused humoral sensitization.

### Chronic toxicity studies in immunodeficient mice

Malignant disease after treatment with Mreg_UKR might, in principle, arise either as consequence of transferring neoplastic cells or as consequence of transferred cells promoting growth of autochthonous tumors.^18^ Neoplastic cells within Mreg_UKR products might originate from the donor, arise during *in vitro* culture or emerge after transfer into the recipient. Not only the therapeutically active cells within a cell product may lead to malignant disease but also cellular contaminants pose a risk of malignant transformation. In theory, immunosuppressive cell therapies might also promote recipient malignancies either by facilitating the growth of autochthonous tumors or by suppressing immune responses against cancerous cells.

To formally assess the risk of M regs causing malignancy or other chronic pathologies, conventional carcinogenicity and chronic toxicity studies were performed in immunodeficient mice. The purpose of this GLP-compliant study was to determine the chronic single-dose toxicity and tumorigenicity of M reg-containing cell preparations. Seventy-five male and 75 female C.B-17-scid mice were divided into three experimental groups (Table 3). Mice in group 1 served as vehicle-only controls. Mice in group 2 received M regs at a BW-adjusted dose (5 × 10^6^ cells/ kg BW) corresponding to the intended treatment dose in humans, whereas mice in group 3 received an eightfold excess cell dose (4 × 10^7^ cells/ kg BW). After treatment with M reg-containing cell preparations, follow-up observations were made over 295 days. These studies showed no abnormal clinical or pathological findings that could be ascribed to M reg exposure. Specifically, clinical and postmortem examination on day 295 after M reg administration revealed no abnormalities of growth, tumor formation, biochemical or hematological disturbances, or any histopathological changes in any of the organs or tissues examined (data not shown).

## Discussion

The manufacture and application of medicinal products is strictly regulated to ensure an appropriate balance of risk and benefit to patients. Under European Union (EU) Law, CBMPs are governed by a legislative framework enacted through EU Regulation 1394/2007/EEC on Advanced Therapy Medicinal Products (ATMPs)^19^ and an amendment of Directive 2001/83/EEC on the Community code relating to medicinal products for human use.^20,21^ At once, this legislation both recognizes the inherent difficulties of studying cell-based therapies as pharmacological agents, but also imposes exacting standards for preclinical characterization of cell products, comparable to those applied to conventional pharmaceuticals.^23^ Complying with these strict regulatory requirements is challenging, especially for academic centers with limited resources^29,30^; moreover, the scientific value of the required safety studies is doubtful, as the Committee for Advanced Therapies (CAT) itself recognizes.^31^

Pharmacokinetic and safety pharmacology studies are performed during nonclinical drug development to assess drug exposure and to identify any possible unwanted drug effects, including rare adverse reactions. Information from such studies is then used to predict safe drug doses for early-phase trials in humans. However, as the results presented in this article illustrate, it is questionable whether preclinical safety testing in animals is a meaningful way of investigating immunologically active CBMPs. A core problem is one of interspecies incompatibility: either a cell product of human origin is tested in animals, which may lack biological relevance, or an analogous animal cell is tested, which does not give direct evidence about the safety of the human cell product. This article highlights the problems of applying animal-into-animal (homologous) and human-into-animal (heterologous) safety testing strategies to CBMPs. In our opinion, preclinical safety testing in animal models provides such poor-quality information that it is largely unhelpful in judging the probable safety profile of CBMPs in patients. Specifically, our confidence in the safety of administering Mreg_UKR to humans is not greatly increased by the safety studies presented here, or by previous studies in mice^10^ and miniature swine,^32^ despite no adverse effects having been identified. Accordingly, we argue that far greater emphasis should be placed on previous clinical experience with identical and closely related cell products when assessing probable clinical safety of novel CBMPs.

### What can be concluded from the absence of adverse reactions in animals?

Few immunologists would contend that animal experiments are not valuable in proof-of-principle demonstrations of the efficacy of new immunotherapies. Why then should we be critical about the value of safety pharmacology and toxicology studies, which use very similar models and techniques as those used for primary and secondary pharmacodynamic studies? One reason is that pharmacodynamic studies aim to detect *particular* biological effects that, in order to be regarded as therapeutically promising, should be relatively large and accrue to all recipients; by contrast, safety pharmacology studies aim to detect *any* adverse biological effects, which may be relatively small or restricted to only a subset of recipients. Self-evidently, proving the absence of detrimental effects requires more sensitive technical and statistical approaches than proving the presence of a beneficial effect.

Before examining the particular case of Mreg_UKR, it is useful to examine the logic of safety testing in animals. In general, it is argued that if a drug has an adverse effect in animal models then it is highly likely to elicit the same adverse reaction in patients; by extension, if a drug does not cause a given adverse reaction in animals, then it is correspondingly unlikely to elicit that reaction in humans. Clearly, this form of analogical reasoning hinges on the *biological relevance* of the animal model to the human system. In the case of chemically synthesized, small-molecule drugs acting at defined pharmacological targets, it may be uncontroversial to accept that its properties in animals are a *correct analogy* for its actions in humans; however, in the case of immunologically active CBMPs, this is often not obviously true. Human and mouse M regs are derived by analogous processes, express very similar phenotypes, and suppress effector T cell function; however, human and mouse M regs are not absolutely alike in phenotype and, whereas iNOS is indispensible for mouse M reg-mediated suppression of T-cell proliferation, it has no proven role in human M reg-mediated suppression.^10,11^ Thus, mouse and human M regs are equivalent cell types, but are not absolutely identical; it follows, for every pharmacological property studied using mouse M regs, it must be shown that human M regs possess a truly analogous property. Likewise, tolerogenic dendritic cell (DC), regulatory T cells and mesenchymal stem cells from mice and humans are divergent in phenotype and effector mechanisms, so the same argument could apply to safety testing of all these cell types.

Another way of interpreting preclinical safety studies is to regard them as a means for drug developers to screen-out potentially harmful cell products at a relatively early stage. This is a pragmatic approach, which concerns itself only with positive evidence of adverse reactions that lead to the conclusion that a product is *likely to be unsafe* in humans. If this is the purpose of preclinical safety studies, then it is crucial to recognize that finding a product is “not unsafe” is not the same as saying that it is “safe”; importantly, it follows that it is not valid to claim that screening for unsafe cell products increases the probable clinical safety of administering cell products found to be “not unsafe” to patients.

It is perhaps counterintuitive to think that extensive preclinical safety testing might not actually increase our confidence in the likely clinical safety of a cell product, but the conclusion can be proven by example. It is striking to note that mouse-into-mouse or human-into-mouse safety preclinical studies would not identify life-threatening acute hemolytic reactions as a consequence of ABO incompatible transfusion of erythrocytes.^33,34^ Similarly, in the field of adoptive transfer of antigen-specific T cells as a cancer therapy, there are many examples of “on-target, off-tumor” adverse effects, especially ocular and central nervous system (CNS) autoimmune reactions, that were not detectable in mice, but caused very serious complications in patients.^35^ Also, in the field of embryonic stem cell transplantation, several groups have produced neural, neuronal or glial progenitors from human embryonic stem cells that were not tumor-forming in animals,^36^ but gave rise to multifocal brain tumors in humans.^37^ These three cases illustrate general reasons for unreliable safety conclusions from preclinical testing, which are: reactions caused by antigens unique to human cell products; reactions caused by antigens unique to human recipients; and, reactions caused by the failure of human tumors to properly engraft in animals. To this general list, we might also add insidious adverse reactions (*e.g.*, immune complex deposition or fibrotic diseases) that may not present within a conventional 18-month toxicology study,^38^ as well as infectious diseases that cannot be transmitted to rodents.

The EMA committee for human medicinal products’ guidelines (CHMP/410869/2006) on human cell-based medicinal products advocates a risk-based approach to safety pharmacology and toxicology studies.^39^ The risk-based approach demands a focused investigation of possible adverse reactions predicted from the known pharmacodynamic and pharmacokinetic properties of that drug. For the most part, immunoregulatory cell types used as CBMPs are naturally occurring components of the immune system; therefore, possible adverse reactions elicited by such cells are predictable because they primarily relate to excessive immunological activity, triggering of unwanted immune responses, an abnormal distribution of cells, or dysregulated cell growth. A detailed risk assessment of Mreg_UKR administration to living-donor kidney transplant recipients has been published elsewhere.^18^ On the basis of this risk assessment, preclinical studies with Mreg_UKR concentrated upon the risk of pulmonary embolic disease and whether M regs cause nonspecific tissue injury at sites of accumulation. No clinically relevant detrimental effect was observed when Mreg_UKR were administered intravenously to NSG mice, either at therapeutic or supratherapeutic doses. Specifically, the cell infusion had no apparent impact on respiratory, cardiac, renal, or neurological function. No gross or microscopic pathology was observed as a result of M reg administration, either at 7 or 295 days postinjection. On the basis of these negative results, there are no safety grounds for terminating development of Mreg_UKR as a CBMP; however, we regard this as a very weak conclusion with no definite implications for the management of patients receiving Mreg_UKR therapy.

So, what useful information can be drawn from safety pharmacology and toxicology studies of CBMPs? To answer this question, we have to establish which properties of our animal models represent correct analogies to the human condition: We are only entitled to draw safety conclusions regarding adverse species-nonspecific effects to which animals and humans are equally susceptible. As already mentioned, the major unwanted secondary pharmacodynamic effects and toxicities of immunologically active CBMPs are most likely to result from their influence over recipient immune responses; unfortunately, it is precisely these complicated and specific immunological interactions that are poorly modeled in animals.^40–42^ On the contrary, adverse reactions that affect systems that are highly conserved between animals and man can be usefully studied in animal models. Pulmonary embolism (PE) of Mreg_UKR is one such example of an analogous adverse reaction, since the diameter of pulmonary vessels and BW-adjusted pulmonary vessel numbers are very similar in all mammals. Accordingly, we can be somewhat reassured by the absence of PE in experimental mice, which implies that PE caused by Mreg_UKR in humans is unlikely at equivalent cell doses.^18^

### How should the potential immunogenicity of CBMPs be assessed?

In the context of solid organ transplantation, treatment with donor-derived M regs has a superior allograft-protective effect compared to recipient-derived M regs.^10^ However, sensitization is an inherent risk of administering allogeneic cells to a patient, which in solid organ transplant recipients could lead to accelerated transplant rejection.^43^ ICH S6 recommendations on the preclinical safety evaluation of biotechnology-derived pharmaceuticals recognize the limitations of studying the immunogenicity of biopharmaceuticals intended for human use in animals.^44^ Specifically, these guidelines acknowledge that induction of an antibody response in animals is not predictive of antibody formation in humans. In our experiments, it would clearly have been meaningless to assess the immunogenicity of human M regs in immunocompetent or immunodeficient mice; therefore, we investigated the potential of allogeneic mouse M regs to exacerbate or attenuate antibody responses in mice receiving an allogeneic heart transplant (*i.e*., a homologous test system). These experiments showed that pretransplant M reg treatment significantly diminished humoral responses against allogeneic cardiac allografts, presumably by suppressing T-cell responses. Nonetheless, the conclusion that allogeneic M reg exposure does not normally elicit alloantibodies in mice cannot be neatly extrapolated to the human situation. Mouse and human M regs, although equivalent cell types, are not absolutely identical in phenotype, so may be differently immunogenic. Additionally, there may be preparation-related factors (*e.g*., dead cell content or manufacturing process-related contaminants) or recipient-related factors (*e.g*., concurrent inflammation or donor–recipient HLA-mismatches) that influence the immunogenicity of infused M regs in patients. Overall, these experiments provided no evidence that administering Mreg_UKR to patients is likely to cause humoral sensitization; however, they do not provide strong support for the conclusion that Mreg_UKR are unlikely to cause sensitization in patients.

ICH S6 recommendations on the preclinical safety evaluation of biotechnology-derived pharmaceuticals advise that toxicity studies in nonrelevant species (*i.e.*, species in which the test substance is not pharmacologically active) may be misleading and are discouraged.^44^ Therefore, we are bound to ask whether our choice of animal models affected the strength of safety conclusions drawn about Mreg_UKR. Specifically, would testing Mreg_UKR in large animal models have provided better evidence of safety? It is fair to assume that human M regs should be more similar to M regs from species with a closer evolutionary relationship to humans than M regs derived from more distantly related species. On this basis, one might expect safety testing of Mreg_UKR in large animals to be generally more informative than rodent experiments. However, experimental group sizes needed to detect clinically relevant adverse effects are often impractical in relevant large animal models. This is certainly true when considering the risk of sensitization: Historical rates of sensitization of patients receiving donor-specific blood transfusion prior to kidney transplantation under cover of azathioprine were 7–16%; therefore, it would necessary to observe 18–42 animals to have a 95% probability of detecting one or more sensitization event. Thus, while large animal experiments certainly provide the most convincing evidence of the efficacy of novel CBMPs, their actual value in toxicological and safety pharmacology studies is much less certain.

### What can be learnt from pharmacokinetic studies in animals?

The EMA committee for human medicinal products’ guidelines on human CBMPs recognize that conventional absorption, distribution, metabolism, and excretion studies are not usually relevant to CBMPs.^22^ However, these same guidelines mandate that pharmacokinetic studies of CBMPs should be carried out to demonstrate tissue distribution, viability, trafficking, growth, phenotype, and any alteration in phenotype due to factors in the tissue environment. After intravenous administration to NSG mice, human M regs partitioned to the lungs, blood, and liver, where a detectable fraction persisted up to 7 days. M regs were not found in spleen or bone marrow, either owing to technical limitations in their detection or because the cells were absent from those tissues.

Tellingly, the distribution of human M regs in NSG mice is not consistent with the distribution of human M regs in humans, since in previous work, we reported that ^111^In-labeled M regs migrated via the blood from the lung to the liver, spleen, and bone marrow. Clearly, studying the distribution and survival of human M regs in NSG mice is unhelpful in predicting the pharmacokinetics of human M regs in patients. There may be many reasons for the discrepant behavior of human M regs in mouse and man, including species-specific differences in soluble mediators and adhesion molecules. Notably, when transferred into mice, human M regs are deprived of tonic M-CSF-stimulation, which is vital for macrophage survival *in vivo*; hence, the lifespan of human M regs in patients may be underestimated from this mouse model.^45^ On the contrary, because NSG mice lack any effective means of rejecting M regs, the NSG mouse model may overestimate the lifespan of allogeneic M regs in an immunocompetent human recipient. The possible use of more sophisticated animal models, such as transgenic mice that better support engraftment of human M regs, does not address the fundamental problem that, without first characterizing the pharmacokinetics of human cells in human recipients, we cannot know whether their distribution and survival in a mouse is an accurate representation. The corollary of not knowing whether human M reg survival in immunodeficient mice is representative of their survival in humans is that only a weak interpretation of the absence of chronic adverse effects observed in our chronic toxicity and tumorigenicity study can be given.

### Are there alternatives to safety pharmacology and toxicology studies in animals?

If, owing to interspecies differences, pharmacological and toxicological studies with mouse or human M regs are liable to produce unreliable safety conclusions, how could the application of Mreg_UKR to patients ever be justified? Elsewhere we have argued that the likely clinical complications of administering cell products to patients are predictable and mitigable.^18^ There is now a substantial literature concerning the administration of therapeutic cell preparations (including regulatory T cells,^46^ tolerogenic DCs,^47^ M regs,^8^ and mesenchymal stem cells)^48^ to patients for a variety of indications. In our opinion, these clinical experiences constitute a much more meaningful basis for assessing the safety profile of other novel cell-based therapies than safety pharmacology and toxicology studies in animals. Explicitly stated, any pharmacological differences between alternative preparations of the same immunoregulatory cell type are generally less important, at least from a safety perspective, than the pharmacological differences between the same immunoregulatory cell type from humans and animals. Therefore, greater confidence in the likely clinical safety of a novel immunoregulatory cell product can be taken from previous studies with similar preparations of the same class of immunoregulatory cell type in humans than from preclinical testing in animals. By extension, we argue that previous clinical experience with immunoregulatory CBMPs is a reliable basis for predicting safe doses of similar CBMPs; furthermore, patient safety information obtained about an immunoregulatory CBMP in one clinical indication is arguably a reliable basis for judging its likely safety profile in other indictions. The obvious conclusion of this argument is that the growing body of clinical safety data relating to immunoregulatory CBMPs largely obviates the need for further safety testing of existing and new CBMPs in animal models.

In a recent article, members of the Paul Ehrlich Institute advocated two possible ways to circumvent the centralized European Marketing Authorization procedure pertaining to ATMPs.^49^ In the first case, they suggest that some ATMPs could be reclassified as transplants or transfusion products, which are less stringently regulated under the Tissues and Cells Directive (Directive 2004/23/EC).^50^ Specifically, cell products which have not undergone substantial manipulations and are intended for homologous use are contenders for classification as non-ATMPs. In the second case, they advocate the use of the Hospital Exemption Rule, a provision made under Article 28 of the EU Regulation on ATMPs for products prepared on a nonroutine basis for a specified patient to be excluded from the central authorization requirements for ATMPs.^19^ Notably, the Hospital Exemption Rule can be used by National Authorities as a regulatory tool for supporting the development and availability of eligible ATMPs, perhaps guiding a particular product into routine manufacturing and then later into central marketing authorization.^49^

In summary, this article presents three lines of argument against the need for further preclinical safety studies of CBMPs in animal models. Firstly, safety pharmacology and toxicity testing of immunologically active CBMPs in heterologous or homologous animal models is questionably relevant to humans and may lead to misleading safety conclusions. Secondly, past clinical experiences with CBMPs are more informative about the probable safety of similar CBMPs in patients than preclinical safety testing in animal models. Thirdly, the burden of conducting preclinical pharmacokinetic, safety pharmacology, and toxicology studies may be great enough to deter development of novel CBMPs. Accordingly, we take a nuanced view of preclinical safety testing of CBMPs in animals: On the one hand, focused investigation of particular adverse reactions in genuinely analogous systems is valuable and, perhaps, there is a case for screening studies with truly novel cell products; on the other hand, it is evident that preclinical safety studies do not generally increase our confidence in the likely safety of CBMPs when administered to patients. Therefore, we contend that there is an urgent need for a debate about the acceptability of trialing novel CBMPs in small-scale exploratory clinical studies with only minimal preclinical safety data, especially when similar CBMPs have been previously applied to patients without adverse effects.

## Materials and Methods

### Manufacture of Mreg_UKR

Leucapheresis products used as starting material for Mreg_UKR generation were produced under a manufacturing license issued by the *Regierung von Oberbayern* (DE_BY_04_MIA_2013_0177/53.2 – ZAB – 2677.1 204). Mreg_UKR were produced under a separate manufacturing license (DE_BY_04_MIA_2013_0187/53.2–2677.1 A 220-O) by Apceth GmbH (Ottobrunn, Germany) according to a proprietary standard operating procedure, which was adapted from previously published protocols.^51^

### Administration of Mreg_UKR to immunodeficient mice

Animal experiments were performed in accordance with permission Nr. 54-2532.1-10/12 granted by the *Regierung von Oberbayern*. NMRI-nude (NMRI-*Foxn1*^*nu*^) mice were obtained from Charles River (Sulzfeld, Germany) and NSG (NOD.Cg-*Prkdc*^scid^ *Il2rg*^*tm1Wjl*^/SzJ) mice were bred in-house. Animals were kept in individually ventilated cages and fed a conventional diet. Recipient mice were anesthetized during Mreg_UKR infusion using 3.6 mg Xylazine plus 27.3 mg ketamine in 1000 μl 0.9% NaCl, given by intraperitoneal injection at 40 μl per 10 g BW. Immediately prior to injection, the concentration of M regs in Mreg_UKR products was adjusted to 10^6^ or 10^7^ viable cells/ml in Ringer’s lactate solution plus 5% human serum albumin. Cell suspensions were injected through a 27-gauge needle into the tail vein of recipient mice over 30–180 seconds.

### Clinical examination of Mreg_UKR recipients

Recipient mice were observed over 7 days for signs of adverse reactions to Mreg_UKR. Changes in BW were monitored. Behavioral, neurological, and autonomic responses to recipient mice were examined using an adaptive version of Irwin’s comprehensive observational assessment.^28^

### Postmortem investigation of NMRI-mice after Mreg_UKR exposure

Recipient mice were killed on day 7 by thoracotomy and exsanguination under anesthesia. Organs were removed, weighed, and prepared for histology. Three micrometer histological sections were cut from paraffin-embedded tissues and stained with hematoxylin and eosin according to standard protocols. Sera were sent for analysis by the Institute of Clinical Chemistry at University Hospital Regensburg using routine diagnostic assays.

### Flow cytometry to detect M regs in tissues from NSG mice

Recipient mice were killed on days 1 to 7 after M reg injection. Human leucocytes were recovered from tissues by physical (spleen and bone marrow) or enzymatic digestion (lung and liver) according to previously described methods.^10^ Single-cell suspensions were passed through a 40-μm mesh (BD Biosciences, Heidelberg, Germany) before enrichment of CD11b^+^ leucocytes by positive human/mouse CD11b magnetic bead selection (Miltenyi, Bergisch-Gladbach, Germany) on an AutoMACS Pro device (Miltenyi).

### Assessing the immunogenicity of mouse M regs in a cardiac transplant model

Abdominal heterotopic heart transplants from C3H donors into BALB/c recipients were performed as previously described in accordance with permission Nr. 54-2532.1-28/09.^10^ Graft rejection was defined as cessation of palpable cardiac contractions with verification by direct inspection of the allograft after laparotomy. Mouse M regs were generated as previously described. Recipient mice either received no additional treatment or received 5 × 10^6^ donor-derived M regs on day 8 prior to transplantation. M regs were resuspended in 1 ml phosphate-buffered saline containing 62 U heparin and administered by slow injection into the tail vein. Sera from all mice were harvested on day 7 posttransplant, and alloantibody levels were measured by flow cytometry cross-match. Briefly, C3H splenocytes were stimulated in overnight culture with concanavalin A. Aliquots of 0.5 × 10^6^ stimulated splenocytes were blocked with mouse FcR blocking reagent (Miltenyi) before incubation for 90 minutes on ice with 50 µl of test serum diluted by 1:500 in Dulbecco’s modified phosphate-buffered saline. Nonimmune sera from naive BALB/c mice were used as a negative control. After incubation, splenocytes were stained with antimouse IgG-FITC, antimouse IgM-APC, and anti-CD3-PE (antibodies from eBioscience, Frankfurt, Germany). For analysis, the CD3^+^ T cell population was gated, and geometric mean fluorescence intensity was determined (FlowJo v7.6.5, Miltenyi).

### Chronic toxicity studies of M reg-containing cell preparations in C.B-17-scid mice

Responsibility for conducting good laboratory practice-compliant chronic toxicity studies was outsourced to a contract research organization (Aurigon Life Science, Gräfelfing, Germany).

### Statistics

Statistical analyses and curve fitting were performed with GraphPad software (La Jolla, CA). Values given in histograms and tables represent mean ± SD. The Mann–Whitney *U*-test was used for nonparametric comparisons between two groups. The Kruksall–Wallis test was used for nonparametric comparisons among three groups.

## Figures and Tables

**Table 1:** Clinical examination of NSG mice treated with Mreg_UKR

	*Vehicle-only*	*10^6^ M regs*	*10^7^ M regs*
Behavioral			
Spontaneous activity			
Sleep	0.0 ± 0.0	0.0 ± 0.0	0.0 ± 0.0
Body position	5.2 P0	5.6 P0	5.6 P0
Locomotor activity	2.0 ± 0.0	2.0 ± 0.0	2.8 ± 1.1
Bizarre behavior	0.0 ± 0.0	0.0 ± 0.0	0.0 ± 0.0
Motor-affective response			
Alley progression (cm)	35.5 ± 14.7	43.9 ± 20.5	45.0 ± 22.3
Transfer arousal	4.0 ± 0.0	4.0 ± 0.0	3.2 ± 1.8
Touch-escape	3.6 ± 0.9	4.0 ± 0.0	4.4 ± 0.9
Positional struggle	2.0 ± 0.0	2.0 ± 0.0	2.0 ± 0.0
Grasp irritability	0.8 ± 0.4	0.8 ± 0.4	1.0 ± 0.7
Provoked biting	3.2 ± 1.8	2.4 ± 2.2	2.0 ± 2.0
Provoked freezing	1.0 ± 0.0	0.8 ± 0.4	1.0 ± 0.0
Finger approach	4.8 ± 1.1	5.2 ± 1.1	5.2 ± 1.1
Positional passivity	3.2 ± 1.1	3.6 ± 1.7	2.4 ± 0.9
Vocalization (events)	0.0 ± 0.0	0.6 ± 0.9	0.6 ± 0.9
Urination (events)	0.2 ± 0.4	0.2 ± 0.4	0.2 ± 0.4
Defecation (events)	2.4 ± 2.1	1.0 ± 1.7	4.0 ± 1.9
Sensory-motor response			
Visual placing	6.0 ± 0.0	6.0 ± 0.0	5.6 ± 0.9
Tail-pinch	2.6 ± 2.3	1.0 ± 0.0	2.4 ± 1.5
Toe-pinch reflex	5.6 ± 0.9	5.6 ± 0.9	6.0 ± 0.0
Corneal reflex	5.2 ± 1.1	4.8 ± 1.1	5.6 ± 0.9
Pinna reflex	2.4 ± 0.9	2.4 ± 0.9	3.6 ± 0.9
Startle	2.8 ± 1.1	5.2 ± 1.8	4.0 ± 1.4
Neurological			
Posture			
Pelvic elevation	4.0 ± 0.0	4.4 ± 0.9	4.4 ± 0.9
Tail elevation	2.0 ± 0.0	2.0 ± 0.0	2.0 ± 0.0
Limb rotation	1.0 ± 0.0	1.0 ± 0.0	1.0 ± 0.0
Muscle tone			
Body tone	6.0 ± 0.0	6.0 ± 0.0	6.0 ± 0.0
Abdominal tone	4.0 ± 0.0	4.4 ± 0.9	5.2 ± 1.1
Limb tone	2.0 ± 0.0	2.0 ± 0.0	2.0 ± 0.0
Grip strength	6.0 ± 0.0	5.6 ± 0.9	4.8 ± 01.8
Wire maneuver	0.4 ± 0.9	0.8 ± 1.8	0.4 ± 0.9
Equilibrium and gait			
Righting reflex	0.0 ± 0.0	0.0 ± 0.0	0.0 ± 0.0
Ataxic gait	0.0 ± 0.0	0.0 ± 0.0	0.0 ± 0.0
Hypotonic gait	0.0 ± 0.0	0.0 ± 0.0	0.0 ± 0.0
Other gait impairment	0.0 ± 0.0	0.0 ± 0.0	0.0 ± 0.0
Total gait incapacity	0.0 ± 0.0	0.0 ± 0.0	0.0 ± 0.0
CNS excitation			
Tremors	0.0 ± 0.0	0.0 ± 0.0	0.0 ± 0.0
Twitches	0.0 ± 0.0	0.0 ± 0.0	0.0 ± 0.0
Convulsions	0.0 ± 0.0	0.0 ± 0.0	0.0 ± 0.0
Autonomic			
Eyes			
Palpebral closure	0.0 ± 0.0	0.0 ± 0.0	0.0 ± 0.0
Exopthalmos	0.0 ± 0.0	0.0 ± 0.0	0.0 ± 0.0
Secretion and excretion			
Lacrimation	1.2 ± 2.7	0.0 ± 0.0	0.2 ± 0.4
Salivation	0.0 ± 0.0	0.0 ± 0.0	0.0 ± 0.0
Diarrhea	0.0 ± 0.0	0.0 ± 0.0	0.0 ± 0.0
General			
Hypothermia	0.0 ± 0.0	0.0 ± 0.0	0.0 ± 0.0
Skin color	4.0 ± 0.0	4.0 ± 0.0	4.0 ± 0.0
Resp. rate	6.0 ± 0.0	6.0 ± 0.0	6.0 ± 0.0
Toxicity			
Acute death (events)	0.0 ± 0.0	0.0 ± 0.0	0.0 ± 0.0

NMRI-nude mice were allocated to three groups of five animals. Mice received injections of either 10^6^ or 10^7^ viable human M regs resuspended in Ringer’s lactate solution plus 5% human serum albumin and 60 U heparin, or were given a vehicle-only injection. Seven days after Mreg_UKR administration, recipient mice underwent clinical examination to identify possible signs of neurological impairment. No significant differences were found. Values represent mean ± SD.

**Table 2:** Postmortem of NSG mice treated with Mreg_UKR

	*Heart (mg)*	*Lungs (mg)*	*Liver (mg)*	*Center kidney (mg)*	*Spleen (mg)*	*Brain (mg)*	*Small intestine (mm)*	*Large intestine (mm)*
Vehicle-only	176.2 ± 39.0	180.2 ± 21.4	1491.0 ± 252.6	229.0 ± 38.5	100.6 ± 27.8	374.2 ± 57.4	455.0 ± 16.9	98.0 ± 6.0
106 M regs	186.0 ± 17.1	202.6 ± 15.4	1588.0 ± 81.8	238.2 ± 18.9	91.6 ± 23.2	337.4 ± 47.1	470.3 ± 28.4	98.3 ± 11.3
107 M regs	189.0 ± 33.3	173.4 ± 18.9	1508.4 ± 141.1	244.6 ± 35.1	132.2 ± 81.8	369.0 ± 40.8	459.6 ± 12.8	100.4 ± 18.0

NMRI-nude mice were allocated to three groups of five animals. Mice received injections of either 10^6^ or 10^7^ viable human M regs resuspended in Ringer’s lactate solution plus 5% human serum albumin and 60 U heparin, or were given a vehicle-only injection. Seven days after Mreg_UKR administration, recipient mice were killed and organ weights or sizes were recorded. No significant differences were found. Values represent mean ± SD.

**Table 3:** Chronic toxicity studies of M reg-containing cell preparations in C.B-17-scid mice

*Group*	*Cohort*	*Cell dose (cells/kg)*	*N*	*Cell density (cells/100 μl)*	*Administration*		*Cotreatment*	*Mortality*		
					*(ml/kg)*	*Route*		♂	♀	Total
1	1	0	13 ♂	—	5	i.v.	None	4/13	9/13	13/26
			13 ♀							
	2	0	12 ♂	—	5	i.v.	None	11/12	7/12	18/24
			12 ♀							
2	1	5 × 10^6^	13 ♂	1 × 10^5^	5	i.v.	None	2/13	6/13	8/26
			13 ♀							
	2	5 × 10^6^	12 ♂	1 × 10^5^	5	i.v.	None	11/12	3/12	14/24
			12 ♀							
3	1	4 × 10^7^	13 ♂	8 × 10^5^	5	i.v.	None	4/13	8/13	12/26
			13 ♀							
	2	4 × 10^7^	12 ♂	8 × 10^5^	5	i.v.	None	10/12	7/12	17/24
			12 ♀							

i.v. intravenously.

Seventy-five males and 75 females C.B-17-scid mice were divided into three experimental groups. Mice in group 1 served as vehicle-only controls. Mice in Group 2 received a bodyweight-adjusted dose of 5 × 10^6^ cells/kg. Mice in group 3 received 4 × 10^7^ cells/kg. After treatment with M reg-containing cell preparations, follow-up observations were made over 295 days.
